# Incidence and Time to Diagnosis of Secondary Malignancies Among Patients With Hairy Cell Leukemia: A Systematic Review and Meta-analysis

**DOI:** 10.7759/cureus.94587

**Published:** 2025-10-14

**Authors:** Akihiro Miyashita, Manasawee Tanariyakul, Chalothorn Wannaphut, Sakditad Saowapa, Kensuke Takaoka

**Affiliations:** 1 Department of Internal Medicine, John A. Burns School of Medicine, University of Hawai'i, Honolulu, USA; 2 Department of Internal Medicine, Texas Tech University Health Sciences Center, Lubbock, USA

**Keywords:** cancer incidence, chemotherapy agents, hairy cell leukemia (hcl), secondary primary cancer, systematic review and meta-analysis

## Abstract

Hairy cell leukemia (HCL) is a rare, indolent, chronic lymphoid neoplasm mainly treated with purine analogs. While some studies report an increased risk of secondary malignancies in patients with HCL, the incidence and the time from HCL diagnosis to the development of secondary cancers remain uncertain. To elucidate the incidence, we conducted a systematic review and meta-analysis. The cumulative incidence of secondary malignancies was 11.2% (95% confidence interval (CI): 8.6-14.3), with a higher incidence observed in patients treated with purine analogs (11.4%, 95% CI: 8.1-15.8) compared with those receiving non-purine analogs (4.8%, 95% CI: 3.6-6.3). The mean time from HCL diagnosis to secondary malignancy development was 100.6 months (95% CI: 56.1-145.0). The mean time varied by treatment group: 74.8 months (95% CI: 61.5-88.0) for non-purine analogs, 116.5 months (95% CI: 31.5-201.5) for purine analogs, and 86.6 months (95% CI: 64.2-109.0) for combination therapies. In conclusion, the choice of initial therapy significantly influences the risk of secondary malignancies, with purine analogs associated with a higher incidence.

## Introduction and background

Hairy cell leukemia (HCL) is a rare, indolent B-cell lymphoproliferative disorder, accounting for approximately 2% of all adult leukemias [1]. It predominantly affects middle-aged to older adults, with a male predominance, and is characterized by pancytopenia (a reduction in red and white blood cells as well as platelets), splenomegaly, and a distinct morphology of malignant cells [1,2]. HCL has a generally favorable prognosis when treated with the appropriate therapies. The introduction of purine analogs, a class of chemotherapy drugs that interfere with DNA synthesis, including cladribine and pentostatin, advanced the treatment of HCL, achieving high complete remission (CR) rates [3].

Despite the efficacy of purine analogs, concerns about long-term outcomes, particularly the risk of developing secondary malignancies, have persisted [4]. Recent cohort studies indicate that patients with HCL may be at increased risk of secondary neoplasms, especially following treatment with purine analogs [5]. Furthermore, a significant association has been observed between prolonged immunosuppression following treatment and the development of secondary hematologic malignancies [3]. While the precise mechanisms underlying this increased risk remain uncertain, it is hypothesized that the immunosuppressive effects of purine analogs, together with prolonged lymphopenia (a reduction in lymphocyte counts) and a disrupted immune environment, may contribute to the emergence of secondary cancers [6-8].

Moreover, it is emphasized that the natural history of HCL, along with the advanced age of typical patients, may further predispose individuals to malignancies such as non-Hodgkin lymphoma, melanoma, and various solid tumors [8,9]. However, the rarity of HCL, in conjunction with a lack of large-scale, randomized clinical trials, has complicated efforts to accurately quantify the true incidence of secondary malignancies and their temporal relationship to the initial HCL diagnosis.

In this systematic review and meta-analysis, we aim to synthesize the available evidence on the incidence and timing of secondary malignancies among HCL patients, stratifying by different frontline therapies, including purine analogs and non-purine-based approaches. By analyzing data from multiple observational studies and recent retrospective cohorts, we intend to provide a comprehensive understanding of the risk factors contributing to the development of secondary malignancies.

This article was previously presented as a meeting abstract at the 66th American Society of Hematology Annual Meeting, San Diego, CA, USA, on December 9, 2024.

## Review

Methods

This meta-analysis adheres to the regulations proposed in Cochrane methodology and uses the reporting strategy outlined in the Preferred Reporting Items for Systematic reviews and Meta-Analyses (PRISMA) [10]. The articles included in this systematic review and meta-analysis were retrieved using PubMed, Google Scholar, and Cochrane CENTRAL databases. The articles included were published from inception to July 2024, utilizing a more detailed search by using medical subject headings (MeSH) and keywords. The conceptual search string used for study search was: ((secondary) OR (second) AND (malignancies) OR (infections) OR (cancers) OR (neoplasms) OR (relapse) AND (patients) OR (individuals) AND (hairy cell leukemia) OR (HCL)).

Eligibility Criteria

The inclusion criteria were as follows: studies that included patients diagnosed with HCL who had undergone treatment; studies that assessed HCL patients treated with purine analogs, non-purine analogs, or a combination of both; studies that reported secondary malignancies after CR of HCL; studies with a minimum of 50 patients; studies involved patients aged 18 years and older; and studies published in English. The exclusion criteria included animal studies, case reports or series, review articles, survey articles, and non-peer-reviewed articles.

Quality Appraisal and Risk of Bias

The Newcastle-Ottawa scale (NOS) was used to assess the methodological quality of the included studies. This scale assesses the quality of studies using three domains: comparability, selection of participants, and reporting of the outcomes [11]. The risk of bias among the included studies was evaluated using the NOS. The NOS consists of a set of questions aimed at assessing the selection of subjects, the comparability of each study, and the outcomes. We used the adapted NOS for cross-sectional studies [11].

Data Extraction

A standardized data extraction was performed using a Microsoft Excel sheet (Microsoft Corp., Redmond, WA, US). The extracted data included study identifiers (name of author and publication year), total sample size, number of patients with secondary malignancies, median of time interval (months) from HCL diagnosis to the development of secondary malignancies, frontline treatments, number of each type of secondary malignancies, and time interval (months) from HCL diagnosis to the development of secondary malignancies of each individual (if available). 

Statistical Analysis

Statistical analysis was performed using R software version 4.5.1 (R Foundation for Statistical Computing, Vienna, Austria). Pooled results were calculated with 95% confidence intervals (CIs) using random-effects models. Cochran’s Q and I² statistic tests were used to assess statistical heterogeneity, with an I² value greater than 50% indicating significant heterogeneity. For studies that did not provide mean and standard deviation, they were estimated using the median, number of patients, and ranges, following the method of Luo et al. [12] and Wan et al. [13].

Results

Literature Search

**Figure 1:**
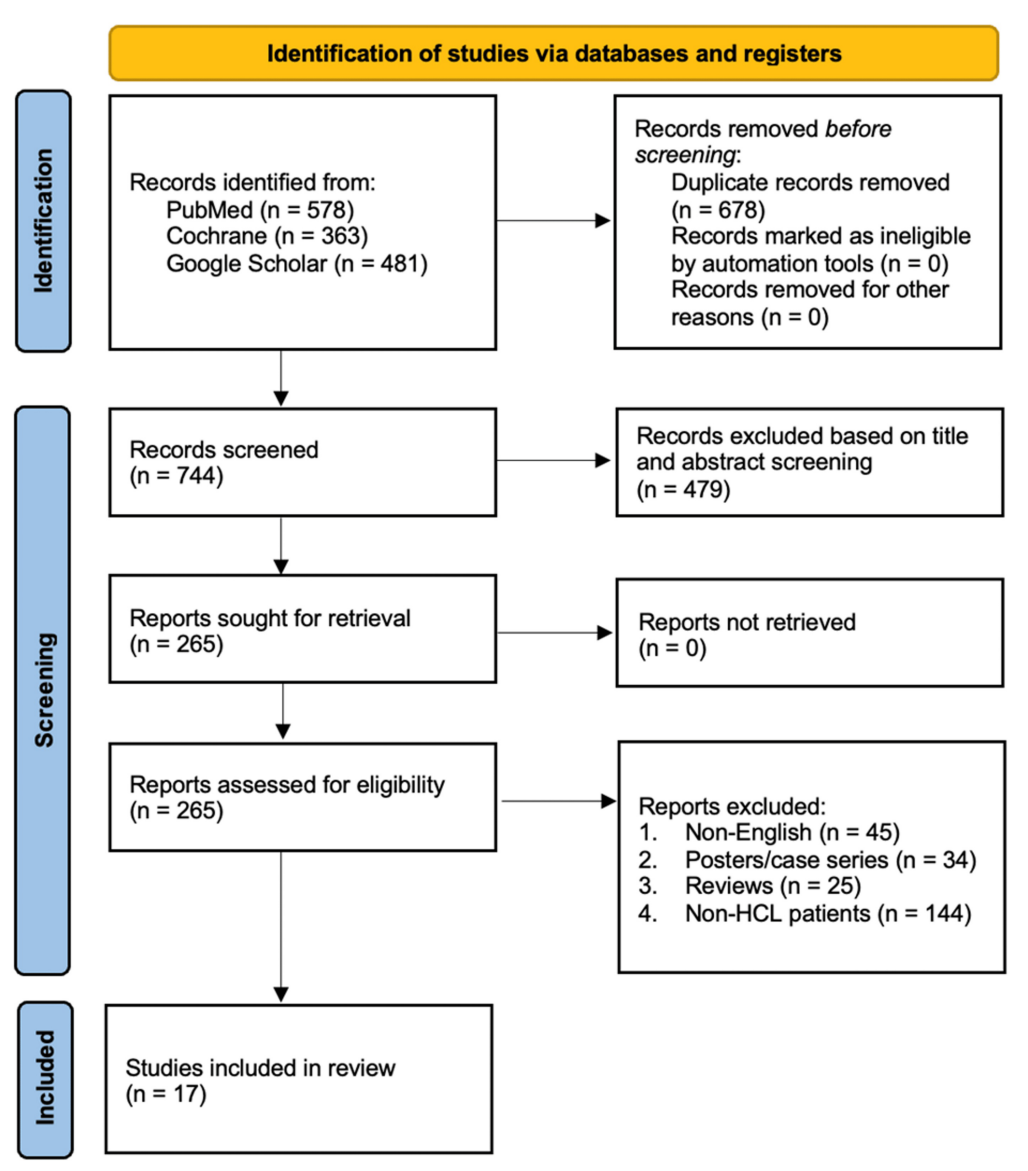
A PRISMA flowchart illustrating the search strategy PRISMA, Preferred Reporting Items for Systematic reviews and Meta-Analyses.

The initial search of the databases identified 1,422 studies. Around 678 articles were duplicates, and 479 irrelevant articles were excluded after reviewing abstracts and titles. The remaining 265 articles were thoroughly assessed based on predetermined eligibility criteria. Consequently, 248 articles were excluded as follows: case series or posters (34), non-English (45), non-HCL patients (144), and reviews (25). A total of 17 studies met the criteria and were included in this review (Figure 1).

Study Characteristics in Secondary Malignancies After HCL Diagnosis

Table 1 presents the characteristics of the included studies by summarizing key details, including total sample size, the number of patients with secondary malignancies, the median time from HCL diagnosis to secondary malignancy, the frontline treatments, including number of patients who received each treatment, number of patients with relapsed HCL, number of patients who received the second-line treatment, and the median follow-up duration. Sample sizes across all studies ranged from 50 to 3,104 patients with a total of 7,837 patients diagnosed with HCL across all studies. The female population was 21.6% of this study, and weighted median age was 55.5 years. Only nine out of the 17 included studies reported the median time and time range from HCL diagnosis to secondary malignancy. The median time from HCL diagnosis to secondary malignancy ranged from 47 months to 301 months. The frontline treatments used included purine analogs such as cladribine and pentostatin, and non-purine analogs, including interferon, rituximab, and splenectomy. Of the 17 included studies, nine used only purine analogs as frontline treatments, while seven studies used a combination of both purine and non-purine analogs. Only one study exclusively used non-purine analogs as the frontline treatment. The number of patients with relapsed HCL varied considerably, ranging from 2 to 131. Similarly, the number of patients who received second-line treatments ranged from 2 to 119. The median follow-up duration ranged from 51 months to 251 months, although a few studies did not report this information.

**Table 1:** Characteristics of included studies *Mean follow-up. †79 relapsed, and five had a lack of response. BSC: Best supportive care; Dx: Diagnosis; F: Female; HCL: Hairy cell leukemia; M: Male; mo.: Month; NR: Not reported; Pts: Patients; R-CHOP: Rituximab, cyclophosphamide, doxorubicin, vincristine, and prednisone; SC: Secondary cancer; Tx: Treatments.

Study	# of pts	M/F	# of pts with SC	Median time (mo.) from HCL Dx to SC (range)	Frontline Tx (# of pts who received each Tx)	# of pts with relapsed HCL	# of pts who received second-line Tx	Median follow-up (mo.)
Benz 2020 [14]	221	170/51	44	68.4 (0.12-210)	Cladribine (221)	53	53	151
Biglari 2022 [15]	50	38/12	1	NR	Cladribine (38) and interferon (4)	9	9	51
Cornet 2014 [9]	487	NR	48	74 (2-375)	Cladribine (281), pentostatin (99), interferon (56), others, including splenectomy alone or purine analog combined with interferon or rituximab (28), and no treatment (23)	131	119	60
Criscuolo 2024 [16]	513	NR	24	59.9 (9-170)	Cladribine (513)	2	2	NR
Else 2009 [3]	233	184/49	28	NR	Cladribine (45) and pentostatin (188)	84^†^	84	192
Federico 2002 [17]	1022	826/196	49	64 (7-206)	Interferon (495), splenectomy (216), pentostatin or cladribine (39), and BSC or single agent chemotherapy (272)	NR	NR	73*
Flinn 2000 [18]	241	193/48	39	NR	Pentostatin (241)	32	NR	112
Getta 2016 [19]	331	260/71	81	NR	Cladribine (220), pentostatin (47), and never treated (64)	NR	111	69
Goodman 2003 [20]	209	167/42	47	NR	Cladribine (209)	76	60	108
Hisada 2007 [21]	3104	2392/712	358	NR	Any chemotherapy (1,509), and other treatment or no chemotherapy (1,595)	NR	NR	78*
Kurzrock 1997 [22]	350	285/65	26	47 (7-220)	Cladribine (126), pentostatin (15), and interferon (146)	NR	NR	72
Madanat 2017 [23]	61	50/11	11	NR	Cladribine (55), pentostatin (1), splenectomy (1), cladribine + rituximab (2), and cladribine + splenectomy (2)	19	17	72
Maloisel 2003 [24]	238	192/46	18	64 (7-267)	Pentostatin (238)	34	32	63.5
Paillassa 2020 [5]	279	NR	59	81 (0-374)	Cladribine (159), pentostatin (49), interferon (40), others, including splenectomy, rituximab, and R-CHOP (19), and no treatment (12)	106	102	127
Rosenberg 2014 [25]	88	63/25	8	301 (178-395)	Cladribine (88)	48	37	251
Saven 1998 [6]	358	NR	27	62 (1-188)	Cladribine (358)	90	66	58
da Silva 2019 [26]	54	51/3	10	NR	Cladribine (36), interferon (6), rituximab (2), splenectomy (7), and no treatment (2)	8	8	108

Incidence of Secondary Malignancies

**Figure 2:**
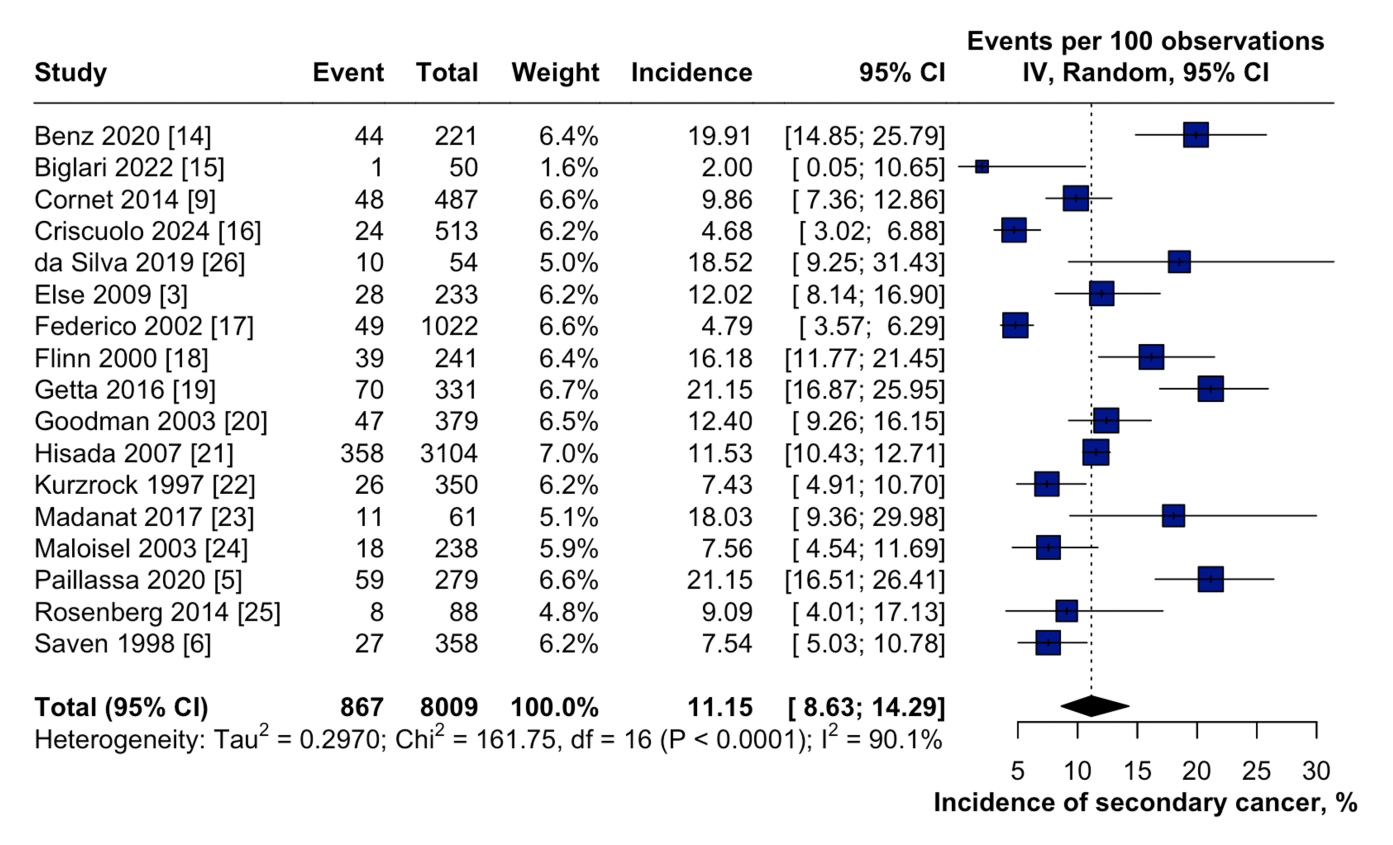
Forest plot showing the cumulative incidence of secondary malignancies in patients with HCL CI: Confidence interval; IV: Inverse variance; HCL: Hairy cell leukemia.

**Figure 3:**
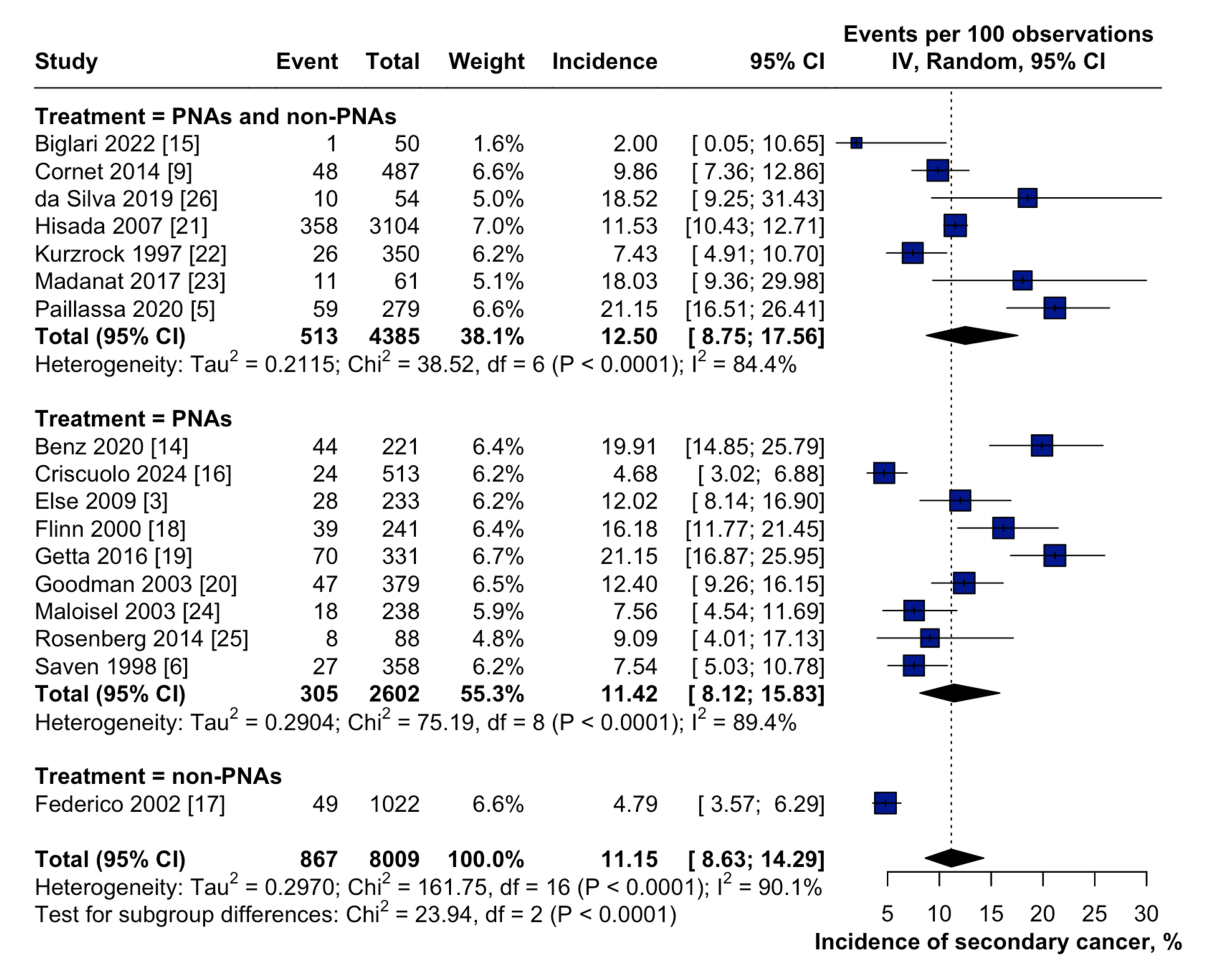
Forest plot showing the cumulative incidence of secondary malignancies in patients with HCL stratified according to the frontline treatment CI: Confidence interval; IV: Inverse variance; HCL: Hairy cell leukemia; PNA: Purine analog.

Data from the 17 included studies showed that the cumulative incidence of secondary malignancies in patients with HCL was 11.2% (95% CI: 8.6-14.3) (Figure 2). A subgroup analysis of these secondary malignancies showed that the incidence was higher when purine analogs were used as frontline treatments for HCL (11.4%, 95% CI: 8.1-15.8). Moreover, the incidence of secondary malignancies in patients receiving both purine analogs and other therapies was 12.5% (95% CI: 8.8-17.6), compared with 4.8% (95% CI: 3.6-6.3) in those who received non-purine analogs (Figure 3). The test for subgroup analysis demonstrated statistically significant differences (p < 0.0001), suggesting that the type of frontline therapy, especially purine analogs, may influence the risk of developing secondary malignancies in patients with HCL.

Out of the 17 studies included in this analysis, 15 provided detailed reports on the types of secondary malignancies. Table 2 provides a comprehensive stratification of the various secondary malignancies occurring after HCL diagnosis, categorized into solid organ tumors, hematological malignancies, and cancers of unspecified type. Prostate cancer was the most frequently reported secondary malignancy, with 187 cases. Skin cancer (both melanoma and non-melanoma skin cancer) and colorectal or anal cancers were also common, with 120 and 79 cases, respectively. Other notable solid organ tumors included lung cancer (68 cases), breast cancer (30 cases), and bladder cancer (29 cases). Regarding hematological malignancies, non-Hodgkin lymphoma was the most common, with 85 cases reported. Other hematologic malignancies included leukemia (18 cases), unspecified hematologic malignancies (15 cases), myelodysplastic syndrome/myeloproliferative neoplasm (13 cases), myeloma (9 cases), and monoclonal gammopathy of undetermined significance/monoclonal gammopathy of clinical significance (8 cases). 

**Table 2:** Types of secondary malignancies occurring after HCL diagnosis ^a^Other includes solid organ malignancies with an incidence of ≤2 patients, including biliary tract cancer, chondrosarcoma, carcinoid tumor, cervical carcinoma, endometrial carcinoma, hepatocellular carcinoma, Kaposi sarcoma, mesothelioma, neuroendocrine tumor, osteosarcoma, ovarian carcinoma, parotid carcinoma, salivary ductal carcinoma, small bowel carcinoma, and vulvar carcinoma. ^b^Other hematological malignancies include lymphoid malignancy and lymphoproliferative disorder. CNS: Central nervous system; HCL: Hairy cell leukemia; MDS: Myelodysplastic syndrome; MGCS: Monoclonal gammopathy of clinical significance; MGUS: Monoclonal gammopathy of undetermined significance; MPN: Myeloproliferative neoplasm.

Secondary malignancies	Number of secondary malignancies
Solid organ tumors	
Prostate	187
Non-melanoma skin cancer	83
Colorectal and anus	79
Lung	68
Melanoma skin cancer	37
Breast	30
Bladder	29
Kidney, pelvis, and urothelial	28
Head and neck	24
Pancreatic	15
Gastric	14
Thyroid	8
Esophagus	5
Soft tissue sarcoma	5
Unknown primary site	5
CNS	4
Other^a^ and unspecified solid organ tumors	40
Hematological malignancies	
Non-Hodgkin lymphoma	85
Leukemia	18
MDS/MPN	13
Myeloma	9
MGUS/MGCS	8
Other hematological malignancies^b^	15
Unspecified types of cancers	21

Time From HCL Diagnosis to the Development of Secondary Malignancies

**Figure 4:**
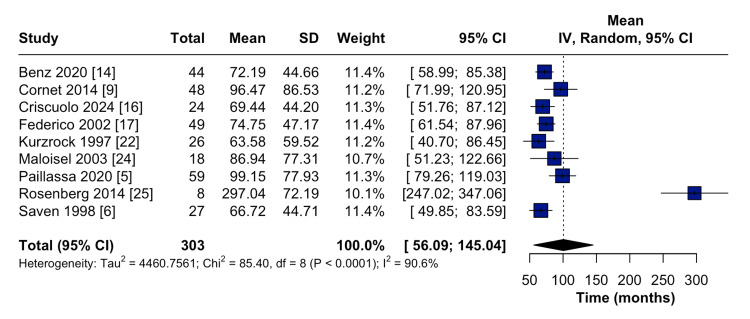
Forest plot showing the pooled mean time from the diagnosis of HCL until the development of secondary malignancies CI: Confidence interval; IV: Inverse variance; HCL: Hairy cell leukemia; SD: Standard deviation.

**Figure 5:**
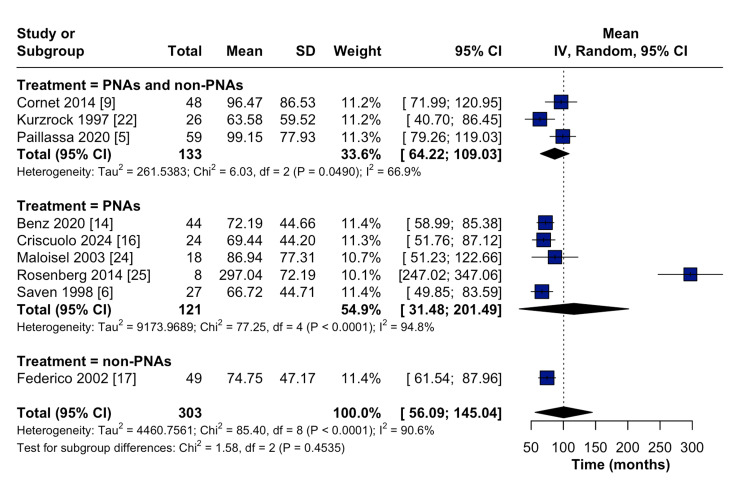
Forest plot showing the pooled mean time from the diagnosis of HCL until the development of secondary malignancies stratified according to the frontline treatment CI: Confidence interval; IV: Inverse variance; HCL: Hairy cell leukemia; PNA: Purine analog; SD: Standard deviation.

Nine out of the 17 studies reported the duration from HCL diagnosis to the development of secondary malignancy. The pooled results showed that the mean time from HCL diagnosis until the development of secondary malignancies was 100.6 months (95% CI: 56.1-145.0) (Figure 4). The subgroup analysis showed that the mean duration until the development of secondary malignancies was 74.8 months (95% CI: 61.5-88.0) for patients who received non-purine analogs as frontline therapies, 86.6 months (95% CI: 64.2-109.0) for patients who received both purine analogs and other treatments as frontline therapies, and 116.5 months (95% CI: 31.5-201.5) for patients who received purine analogs as frontline therapies (Figure 5). This finding suggests that patients who received purine analogs had a longer time to the development of secondary malignancies compared with those who received non-purine analogs or combination therapies.

Quality Appraisal

All the included studies were evaluated using NOS and were found to have good methodological quality (Table 3). The risk of bias across studies was negligible, supporting the quality of the pooled results.

**Table 3:** Methodological quality using the Newcastle-Ottawa Scale AHQ criteria AHQ: Assessment of heterogeneity and quality.

Study name	Selection	Comparability	Outcomes	AHQ standard
Benz 2020 [14]	3	2	3	Good
Biglari 2022 [15]	3	2	2	Good
Cornet 2014 [9]	3	1	3	Good
Criscuolo 2024 [16]	3	2	2	Good
Else 2009 [3]	3	1	2	Good
Federico 2002 [17]	3	2	3	Good
Flinn 2000 [18]	3	1	3	Good
Getta 2016 [19]	3	2	2	Good
Goodman 2003 [20]	3	1	2	Good
Hisada 2007 [21]	3	2	3	Good
Kurzrock 1997 [22]	3	1	3	Good
Madanat 2017 [23]	3	2	2	Good
Maloisel 2003 [24]	3	1	2	Good
Paillassa 2020 [5]	3	2	3	Good
Rosenberg 2014 [25]	3	1	3	Good
Saven 1998 [6]	3	2	2	Good
da Silva 2019 [26]	3	1	3	Good

Discussion

In this meta-analysis, we concluded that the cumulative incidence of secondary malignancies was 11.2%, and treatment with purine analogs such as cladribine became a greater risk of secondary malignancy compared with treatment with non-purine analogs in patients with HCL. Additionally, the time from diagnosis of HCL to the development of secondary cancer was notably long, with a mean duration of more than eight years across all treatment groups. This indicates that patients with HCL, particularly those treated with purine analogs, are at a prolonged risk for secondary cancer, and we may need long-term surveillance for those patients.

The etiology of secondary cancer remains to be investigated, but one of the possible causes is the immunosuppressive effect caused by purine analogs. They induce a marked decrease in CD4 cells for several years, which leads to prolonged immunosuppression [27]. When the immune system is compromised, its ability to detect and eliminate precancerous cells is reduced, increasing the likelihood of cancer development. Immunosuppression might also be caused by alterations in the immune system secondary to HCL itself [28], making it difficult to conclude that purine analogs are a definitive contributing factor. Another possible cause is direct DNA damage by purine analogs and other antineoplastic agents. They work by interfering with the synthesis of DNA and RNA in cells, leading to apoptosis of tumor cells. While this is an effective mechanism for treating cancers, it can also cause mutations in healthy cells.

Our analysis elucidated that prostate cancer, the second most common cancer among men worldwide, was the most frequently observed secondary malignancy, followed by non-Hodgkin lymphoma and non-melanoma skin cancer [29]. The male predominance of HCL and the recent widespread use of prostate-specific antigen testing may be contributing factors to the higher incidence of prostate cancer. Additionally, HCL and most non-Hodgkin lymphomas share a common origin, as both arise from B lymphocytes, which may help explain the high incidence of non-Hodgkin lymphomas.

Several studies reported the risk for secondary malignancies in patients with other hematological malignancies treated with cytotoxic chemotherapy. Diffuse large B-cell lymphoma (DLBCL) is the most common type of lymphoma worldwide, which is usually treated with rituximab-containing immunochemotherapy such as rituximab, cyclophosphamide, doxorubicin, vincristine, and prednisone (R-CHOP). One retrospective analysis showed that the incidence of secondary cancer in patients with DLBCL treated with rituximab-containing chemotherapy was 7.1%, and the median time to diagnosis of secondary malignancies was 30 months, which was much shorter than that of patients with HCL in the present study [30]. One of the possible reasons for this difference is that secondary cancers in patients with HCL may primarily result from the immunosuppressive effects caused by purine analogs, whereas in patients with DLBCL, secondary cancers are more likely driven by direct DNA damage from multi-agent chemotherapy such as R-CHOP, leading to the earlier development of secondary malignancies. Furthermore, hematologic malignancies accounted for only 5.8% of all secondary cancers in patients with DLBCL, which is remarkably lower than the 17.8% observed in patients with HCL. A further investigation is warranted to elucidate this phenomenon.

Limitations

The limitation of this study is that it is unable to determine whether the observed cancers are de novo, related to the underlying disease, or a result of treatment. Although we observed the cumulative incidence of secondary cancer in patients with HCL of 11.2%, we are unable to determine whether HCL independently increases the risk for secondary neoplasms, as we did not compare with a cohort of healthy individuals. Given that the median age of HCL diagnosis is between 50 and 55 years, a period when cancer incidence naturally rises, similar outcomes might be observed in a healthy cohort [31]. Furthermore, our analysis lacks data on the number of cladribine treatment cycles, making it difficult to establish a relationship between cumulative chemotherapy dosing and the risk of secondary malignancies. For instance, etoposide, a topoisomerase II inhibitor, has a correlation between the risk of secondary leukemia and the administered cumulative dose of etoposide [32]. Most patients with HCL treated with cladribine achieve CR in 80% to 90% of cases after one cycle of chemotherapy, with a median duration of response of approximately 10 years [4,33,34]. Establishing the relationship between cumulative doses of cladribine and secondary cancer risk would be beneficial, especially for young individuals, as long-term risks must be carefully considered when making treatment decisions.

Another limitation of this meta-analysis is the variability in follow-up periods across the included studies, which may impact the observed incidence of secondary malignancies in patients with HCL. In our analysis, the median time from HCL diagnosis to the development of secondary cancer was 100.6 months, suggesting that some studies may not have had a sufficiently long follow-up period to accurately capture the true incidence. Studies with shorter follow-up durations may underestimate secondary cancer risk, as malignancies developing beyond their observation period would not be accounted for. This bias is particularly concerning in the context of secondary cancers, which often emerge many years after HCL diagnosis.

## Conclusions

This systematic review and meta-analysis included 17 studies comprising 7,837 patients with HCL. The pooled cumulative incidence of secondary cancer was 11.2%. Subgroup analysis showed that patients treated with purine analogs had a higher incidence of secondary cancer compared with those who received non-purine analog therapies (11.4% vs. 4.8%). The most frequent secondary malignancies were prostate cancer, followed by skin cancer and colorectal or anal cancer. The mean time from HCL diagnosis to the development of secondary cancer exceeded eight years overall, with six years for patients treated with non-purine analogs and nine years for those treated with purine analogs. These findings suggest that patients with HCL, particularly those exposed to purine analogs, remain at elevated risk for secondary cancers over the long term, underscoring the need for extended surveillance.
